# Development and external validation of a nomogram for choosing postoperative adjuvant therapies in uterine sarcoma patients using real-world data

**DOI:** 10.3389/fonc.2025.1609721

**Published:** 2025-09-10

**Authors:** Ling Li, Weili Tao, Ze Ouyang

**Affiliations:** ^1^ Department of Medical Ultrasonic, Affiliated Cancer Hospital of Zhengzhou University & Henan Cancer Hospital, Zhengzhou, China; ^2^ Department of Medical Oncology, Hubei Cancer Hospital, Tongji Medical College, Huazhong University of Science and Technology, Wuhan, China

**Keywords:** SEER, nomogram, uterine sarcoma, postoperative adjuvant therapy, external validation

## Abstract

**Objective:**

This study aimed to develop and validate a prognostic nomogram to identify uterine sarcoma (US) patients who may not require adjuvant therapy after surgery, based on data from the Surveillance, Epidemiology, and End Results (SEER) database and an external Asian cohort.

**Methods:**

Data from eligible uterine sarcoma patients in the USA (*n* = 1,626) who met the criteria of this study were collected from the SEER database and randomly divided into a training cohort (*n* = 1,138) and an internal validation cohort (*n* = 488). Multivariate Cox regression, Lasso regression, and crossvalidation were performed to select the optimal variables associated with survival. A nomogram-based model was then constructed to stratify the recurrence risk thresholds for the assessed patients. An external dataset from a separate cohort at our hospital (*n* = 90) was used to validate the accuracy and specificity of the nomogram model in discriminating patient risks, utilizing the consistency index (C-index), receiver operating characteristic (ROC) curves, calibration curves, and decision curve analysis (DCA).

**Results:**

Using the aforementioned classification aggregation methods, analysis of the training cohort identified diagnostic age, Fédération Internationale de Gynécologie et d'Obstétrique (FIGO) stage, grade, tumor size, and peritoneal cytology as independent predictors of overall survival (OS). The subsequent risk model demonstrated that patients with a threshold below 55 had a 10-year survival rate exceeding 80%, suggesting they may not require postoperative adjuvant therapy. Internal validation confirmed the reliability of this multiparameter model, as evidenced by a C-index of 0.77 and ROC AUC values of 0.812, 0.824, and 0.839 for 1-, 3-, and 5-year OS, respectively. Similarly, accuracy and specificity were confirmed by the external validation cohort, with a C-index exceeding 0.83, reaching a peak of 0.9, and ROC AUC values greater than 0.876. These results highlight that the stratified thresholds displayed by our nomogram outperformed FIGO staging in identifying low-risk recurrence patients.

**Conclusion:**

Our constructed multiparameter nomogram model appears to be superior to the FIGO staging system in identifying low-risk patients who do not require adjuvant therapy after surgery, although prospective data are required for further validation.

## 1 Introduction

Uterine sarcomas are rare mesenchymal malignant tumors; their incidence among uterine corpus cancers is only three to seven cases per 100,000 (1). There are several pathological types of uterine sarcoma, including leiomyosarcoma (LMS), low-grade endometrial stromal sarcoma (LG-ESS), high-grade endometrial stromal sarcoma (HG-ESS), undifferentiated endometrial sarcoma (UES), rhabdomyosarcoma (RMS), and adenosarcoma (MA), among others. Patients with different pathological types have distinct prognoses and treatments (2). Up to 58.8% of patients are diagnosed with malignant sarcoma through postoperative pathology, and clinical and radiological criteria often make it challenging to differentiate leiomyomas from malignant uterine tumors (3, 4).

Nowadays, surgical resection remains the most effective curative treatment. Patients typically undergo individualized therapy, which may include surgery and follow-up chemoradiotherapy (5). Omura et al. enrolled 156 postoperative uterine sarcoma (US) patients with stage I or stage II disease and administered Adriamycin for 6 months or no further treatment.

Unfortunately, no significant differences were observed in progression-free survival (PFS) and overall survival (OS) (6). In the SARCGYN phase III study, Pautier et al. compared polychemotherapy followed by pelvic radiotherapy and radiotherapy alone in 81 patients. There was no statistical significance in 3-year OS, and the combination treatment was associated with a higher incidence of grades 3–4 toxicity (7). Therefore, we aim to build a prognostic model to more accurately identify patients who do not require adjuvant therapy after surgery and those who need aggressive adjuvant therapy.

Zivanovic et al. found that, in patients with uterine leiomyosarcoma, American Joint Committee on Cancer and Fédération Internationale de Gynécologie et d'Obstétrique staging had their advantages in predicting the prognosis of patients with early- or late-stage disease, but neither was accurate enough. Inaccurate prognostic judgments can influence treatment choice, and the nomogram showed a more pronounced predictive advantage than traditional staging (8). A nomogram can more accurately assess the course of the disease and help physicians better identify patients who may achieve prolonged survival from postoperative adjuvant therapy (9). The increasing application of nomograms for predicting survival outcomes is becoming important across various types of tumors (10–14).

Thus far, some studies have developed nomograms for patients with uterine sarcoma. Cao et al. identified age, race, marital status, tumor primary site, stage, and grade as variables for building a nomogram (15). Li et al. constructed a nomogram using age at diagnosis, surgery status, Surveillance, Epidemiology, and End Results (SEER) stage, American Joint Committee on Cancer (AJCC) stage, histological grade, chemotherapy, insurance record, tumor size, race, radiotherapy, and marital status (16).

This study explored the impact of lymph node dissection during surgery, postoperative adjuvant treatment, and demographic factors on patient survival. We used only four variables to build a model that could predict the survival probability of 1-, 3-, and 5-year overall survival in Asians.

## 2 Materials and methods

### 2.1 Date sources

Our research was based on data from public databases and retrospective hospital records. The SEER database is a publicly accessible, authoritative tumor registry widely used in clinical research, containing information on millions of cancer patients in the USA, including age at onset, age at death, primary tumor site, surgical details, treatment information, and demographic characteristics. In this study, we extracted patient data from the SEER database using SEER*Stat software (version 8.4.0.1; http://seer.cancer.gov/), account number 15006-Nov2021, for cases diagnosed between 1 January 2010 and 31 December 2019. Additionally, we obtained an external validation set from Tongji Hospital, Huazhong University of Science and Technology (THAHUST), using the hospital’s electronic medical record system, for cases diagnosed between 1 January 2009 and 31 December 2018.

### 2.2 Patient selection

We retrieved a total of 1,626 available cases from the SEER database. We also identified 90 cases for an external validation set in THAHUST’s electronic medical record system. Patient data were collected based on the following criteria: (1) download data from the SEER database according to ICD-O-3.2 morphological code, including ICD-O-3 8714/3: perivascular epithelioid cell tumors, ICD-O-38896/3: mycinous leiomyosarcoma, ICD-O-3 8805/3: undifferentiated uterine sarcoma, ICD-O-38900/3: rhabdomyosarcoma, ICD-O-3 8890/3: leomyosarcoma, ICD-O-3 8891/3: epithelial leiomyosarcoma, ICD-O-3 8930/3: high-grade endometrial stromal sarcoma, ICD-O-3 8931/3: low-grade endometrial stromal sarcoma, ICD-O-3 8933/3: rhabdomyosarcoma. It should be noted that carcinosarcoma should not be included.

According to the WHO Classification of Tumors of the Female Genital Organs (fourth edition, 2014), carcinosarcoma is clearly classified as “metaplastic carcinoma” and placed under the category of endometrial cancer rather than mesenchymal tumors (17). (2) All patients were identified by pathological examination as first primary tumors, (3) underwent surgical resection, (4) had complete postoperative pathological data and general clinical characteristics, and (5) had complete follow-up data. The exclusion criteria were (1) not being the first or only primary malignancies and (2) patients with insufficient information.

### 2.3 Statistical analysis

Patient survival information was quantified using OS, which is defined as the time from the surgical pathological diagnosis to the date of last follow-up or death from any cause. The clinicopathological factors we collected, which may be related to patient survival, included age at diagnosis, FIGO stage, radiotherapy, chemotherapy, race, marital status, pathology, tumor grade, peritoneal cytology, pelvic lymph node log odds of positive lymph node (LODDS) grade, paraaortic lymph node LODDS grade, and tumor size. For constructing and validating the nomogram, the 1,626 patients collected from the SEER database were randomly assigned as the training set (*n* = 1,138) and internal validation set (*n* = 488) in a 7:3 split ratio, while a total of 90 cases collected from THAHUST served as an external validation set. Continuous variables, including age at diagnosis, tumor size, and lymph node LODDS grade, were analyzed using R Studio 4.2.2 (http://www.rstudio.com/) with the “survivalROC” and “survminer” packages to obtain optimal cut-off values in the training set. Categorical variables were presented as numbers with percentages. The demographic and clinical characteristic distributions between the training and validation sets were compared using the Chi-square test with SPSS. Modeling variables were obtained by univariate and multivariable Cox regression, as well as Lasso regression, and prognostic models based on the identified independent prognostic factors.

We calculated the consistency index (C-index), the area under the time-dependent receiver operating characteristic (ROC) curve (AUC) to evaluate the predictive accuracy of the nomogram. The predictive accuracy (discrimination) of a nomogram is measured by the C-index, which quantifies the level of agreement between the predicted probability and the actual probability of an event of interest occurring (18).

A C-index and AUC value above 0.90 indicate high predictive power, while values between 0.71 and 0.90 suggest moderate discrimination. We plotted a calibration curve to evaluate the model and assess potential overfitting using 1,000 bootstrap resamples. The DCA provides a clear answer to which model will bring the most significant clinical benefit, on average, to the right patient, comparing the constructed nomogram with the net clinical benefit of FIGO staging by the DCA.

**Figure 1:**
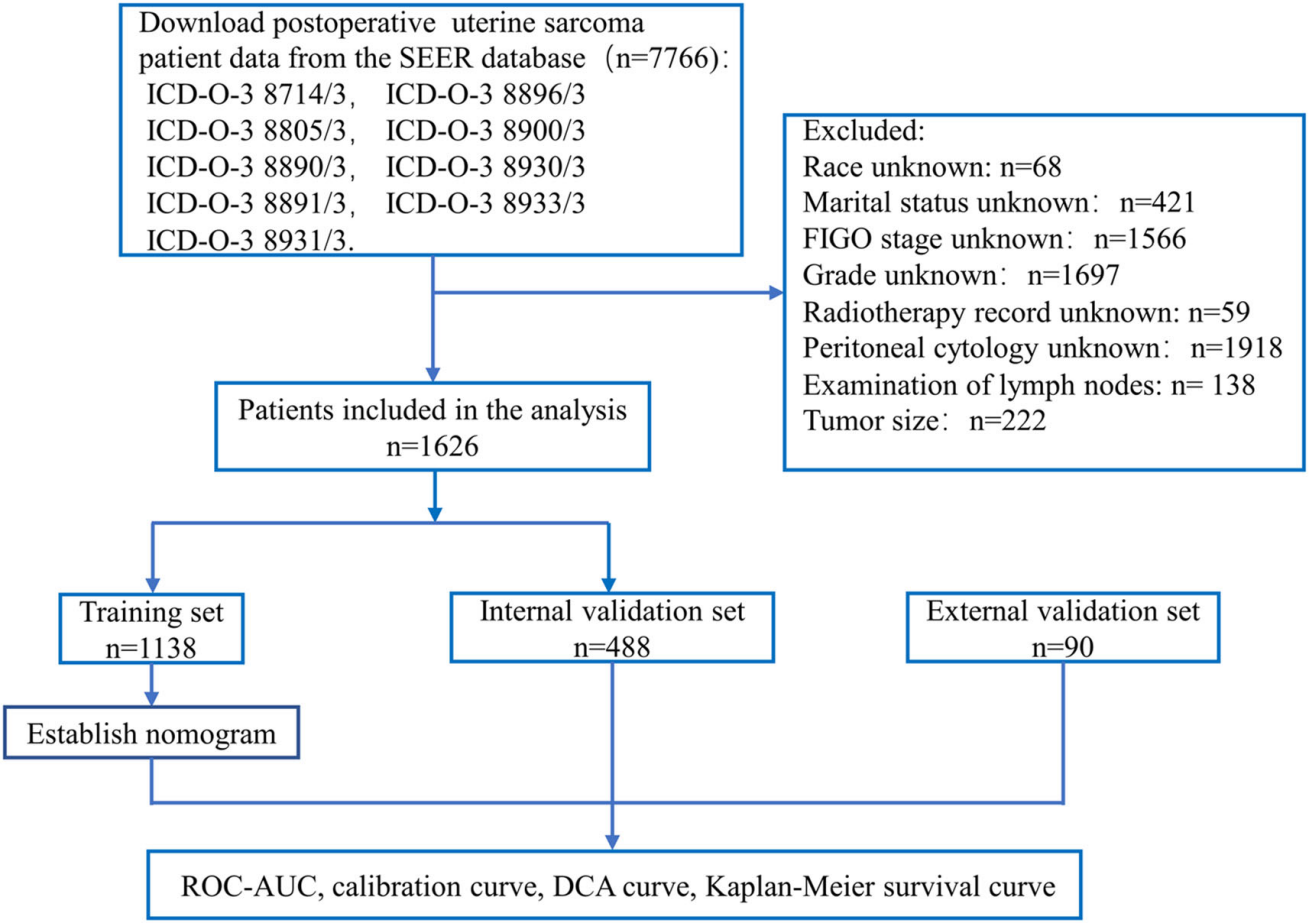
Flow chart for constructing a nomogram based on SEER database data and validating model performance.

Finally, the total score of each patient was calculated based on the variable scores in the nomogram. The patients were stratified into a high-risk group, a medium-risk group, and a low-risk group based on the 5-year survival probability. The Kaplan–Meier curve for OS was plotted according to risk grouping, and log-rank tests were conducted in the training and validation sets. The research process is shown in **Figure 1**.

## 3 Results

### 3.1 Optimal cut-off values result

We got the optimal cut-off values based on the training set in **Table 1**.

**Table 1:** The best cut-off values for continuous variables obtained using R software.

Variables	Cut-off value	Minimum	Maximum
Age (year)	52	16	92
Tumor size (mm)	81	3	986
Pelvic lymph node LODDS grade	− 0.9542425	− 2.021189299	1.431363764
Paraaortic lymph node LODDS grade	− 0.4771213	− 1.799340549	1.113943352

### 3.2 Patient characteristics

Collated clinical and demographic baseline characteristics for training sets, internal validation sets, and external validation sets are presented in **Tables 2**, **3**. In the data from the SEER database, the median age at diagnosis was 55 years (quartile, 48–64 years). About half of the patients were diagnosed with stage I (55.54%).

**Table 2:** Summary of patient data collected from the SEER database.

Variables	Total (*n* = 1,626)	Training set (*n* = 1,138)	Internal validation set (*n* = 488)	*p-*value
Status				
Alive	834	587	247	
Dead	792	551	241	
Survival time (months; median [QR])	34.5 [13.00, 65.00]	34.5 [13.75, 64.00]	34.5 [12.00, 69.75]	
Age (*n* [%]; median [QR])	55 [48, 64]	55 [48, 65]	55 [48, 64]	0.606
< 52 years	616 (37.88%)	426 (37.43%)	190 (38.93%)	
≥ 52 years	1,010 (62.12%)	712 (62.57%)	298 (61.07%)	
Race (*n* [%])				
White	1,176 (72.32%)	830 (72.93%)	346 (70.90%)	0.6373
Black	276 (16.97%)	191 (16.78%)	85 (17.42%)	
Other	174 (10.70%)	117 (10.28%)	57 (11.68%)	
Married status (*n* [%])				
No	388 (23.86%)	268 (23.55%)	120 (24.59%)	0.6984
Yes	1,238 (76.14%)	870 (76.45%)	368 (75.41%)	
FIGO stage (*n* [%])				
I	903 (55.54%)	635 (55.80%)	268 (54.92%)	0.1403
II	241 (14.82%)	172 (15.11%)	69 (14.14%)	
III	41 (2.52%)	22 (1.93%)	19 (3.89%)	
IV	441 (27.12%)	309 (27.15%)	132(27.05%)	
Pathology type (*n* [%])				
LG-ESS	287 (17.65%)	204 (17.93%)	83 (17.01%)	0.4168
LMS	756 (46.49%)	530 (46.57%)	226 (46.31%)	
HG-ESS	294 (18.08%)	214 (18.80%)	80(16.4%)	
UES	61 (3.75%)	41 (3.60%)	20 (4.10%)	
RMS	25 (1.54%)	14 (1.23%)	11 (2.25%)	
MA	203 (12.48%)	135 (11.86%)	68 (13.93%)	
Tumor size (*n* [%])				
< 81 mm	762 (46.86%)	536 (47.10%)	226 (46.31%)	0.812
≥ 81 mm	864 (53.14%)	602 (52.90%)	262 (53.69%)	
Grade (*n* [%])				
Grade 1	170 (10.46%)	117 (10.28%)	53 (10.86%)	0.0488
Grade 2	407 (25.03%)	294 (25.83%)	113 (23.16%)	
Grade 3	324 (19.93%)	207 (18.19%)	117 (23.98%)	
Grade 4	725 (44.59%)	520 (45.69%)	205 (42.00%)	
Peritoneal cytology (*n* [%])				
Negative	651 (40.04%)	453 (39.81%)	198 (40.57%)	0.7102
Positive	71 (4.37%)	47 (4.13%)	24 (4.92%)	
Unknown	904 (55.60%)	638 (56.06%)	266 (54.51%)	
Radiotherapy (*n* [%])				
No	1,367 (84.07%)	961 (84.45%)	406 (83.20%)	0.5774
Yes	259 (15.93%)	177 (15.55%)	82 (16.80%)	
Chemotherapy (*n* [%])				
No	961 (59.10%)	681 (59.84%)	280 (57.38%)	0.3835
Yes	665 (40.90%)	457 (40.16%)	208 (42.62%)	
Pelvic LODDS (*n* [%])				
≤ 0.9542	470 (28.91%)	347 (30.49%)	123 (25.20)	0.0361
≥ − 0.9542	1,156 (71.09%)	791 (69.51%)	365 (74.80%)	
Paraaortic LODDS (*n* [%])				
≤ 0.4771	305 (18.76%)	221 (19.42%)	84 (17.21%)	0.3293
≥ − 0.4771	1,321 (81.24%)	917 (80.58%)	404 (82.79%)	

**Table 3:** Summary of patient data collected from THAHUST.

Variables	External validation set (*n* = 90)
Status	
Alive	48 (53.3%)
Dead	42 (46.7%)
Survival time (months)	
Median (QR)	56.5 [16.75, 108]
Age (*n* [%]; median [QR])	47 [39, 55.25]
Race (%)	
< 51	55 (61.11%)
≥ 52	35 (38.99%)
Other	90 (100.0%)
Married status (%)	
No	3 (3.3%)
Yes	12 (13.3%)
FIGO stage (*n* [%])	
I	51 (56.67%)
II	11 (12.22%)
III	16 (17.78%)
IV	12 (13.33%)
Pathology (%)	
LG-ESS	39 (43.3%)
LMS	21 (23.3%)
HG-ESS	13 (14.4%)
UES	12 (13.3%)
RMS	1 (1.1%)
MA	4 (4.4%)
Tumor size (*n* [%])	
< 81 mm	62 (68.89%)
≥ 81 mm	28 (31.11%)
Grade (%)	
Grade 1	41 (45.6%)
Grade 2	22 (24.4%)
Grade 3	16 (17.8%)
Grade 4	11 (12.2%)
Peritoneal cytology (%)	
Negative	84 (93.3%)
Positive	6 (6.7%)
Radiotherapy (%)	
No	73 (81.1%)
Yes	17 (18.9%)
Chemotherapy (%)	
No	30 (33.3%)
Yes	60 (66.7%)
Pelvic LODDS (%)	
≤ 0.4771	12 (13.3%)
≥ − 0.4771	78 (86.7%)

Leiomyosarcoma has the highest incidence (46.49%), followed by endometrial stromal sarcoma (35.73%). A total of 40.9% of the patients received postoperative chemotherapy, and only 15.93% received radiotherapy. Only 4.37% of patients were positive for peritoneal cytology. In THAHUST, the median age at diagnosis was 47 years (quartile, 39–55.25 years old), and it had a longer median survival time (SEER: 34.5 vs. THAHUST: 56.5). THAHUST data also showed a higher proportion of patients receiving postoperative adjuvant therapy.

### 3.2 Lasso regression analysis and Cox regression analysis results

**Figure 2:**
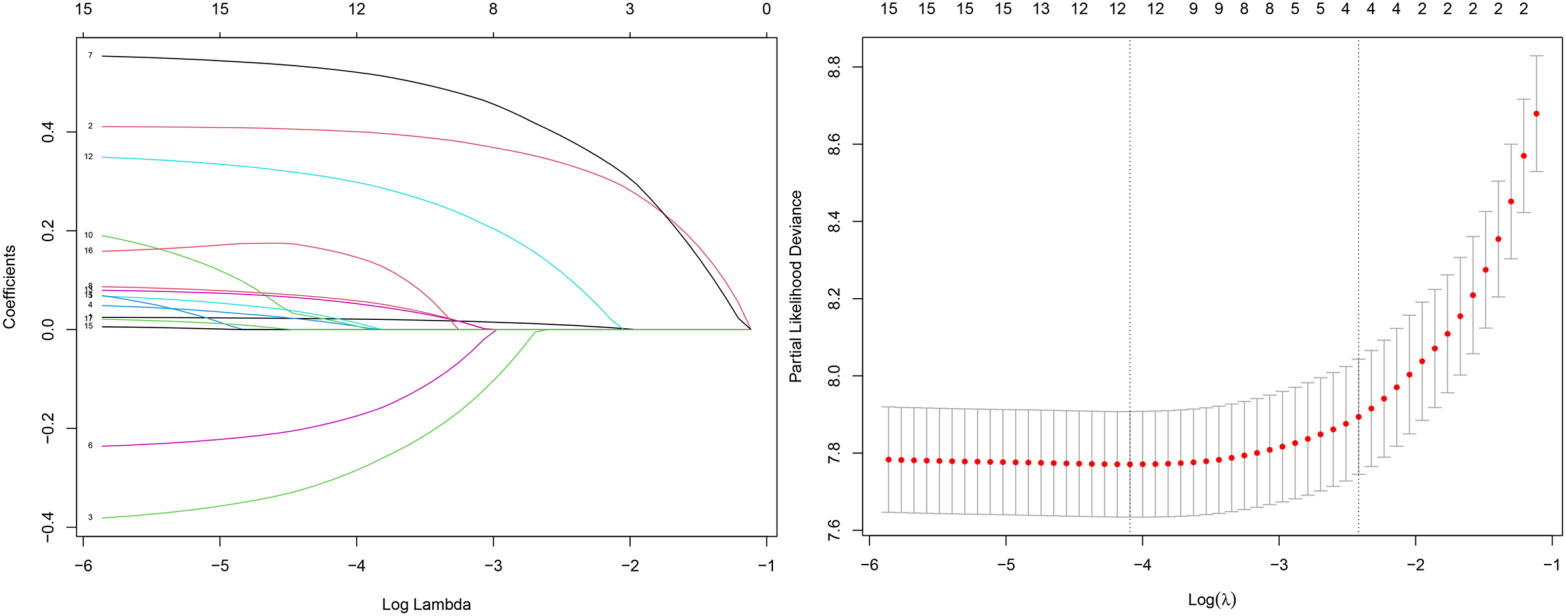
Lasso regression results.

We first explored the optimal variables through Lasso regression, which identified four variables: age at diagnosis, FIGO stage, grade, and tumor size. The results of the Lasso regression are presented in **Figure 2**.

According to univariate Cox regression analysis, eight variables were identified with a *p*-value < 0.05, including age at diagnosis, FIGO stage, chemotherapy, race, grade, peritoneal cytology, tumor size, and pathology. According to the multivariable Cox regression analysis, age at diagnosis, FIGO stage, race, grade, peritoneal cytology, pathology, and tumor size were independent prognostic factors. Age at diagnosis (hazard ratio (HR) = 1.625 vs. age younger than 52 years, *p* < 0.001), FIGO stage (stage II, HR = 1.93 vs. FIGO stage I, *p* < 0.001; stage III, HR = 1.899, *p* = 0.024; stage IV, HR = 3.463, *p* < 0.001), race being Black (HR = 1.513, *p* < 0.001), tumor grade (grade 3, HR = 2.903 vs. grade 1, *p* < 0.001; grade 4, HR = 2.855, *p* < 0.001). Consistent with other studies, Black race was an independent risk factor for uterine sarcoma (16). The results of univariate and multivariable Cox regression analyses are presented in **Table 4**.

**Table 4:** Results of selected variables analyzed by univariate and multivariable Cox regression analyses.

Variables		Univariate analysis		Multivariable analysis		
		HR (95% CI)	*p*-value	HR (95% CI)		*p*-value
Age (*n* [%])						
< 52		Reference		Reference		
≥ 52		2.265 (1.863, 2.754)	**<** 0.001	1.625 (1.330, 1.985)		**<** 0.001
FIGO stage (*n* [%])						
I	Reference			Reference		
II	2.307 (1.800, 2.957)		**<** 0.001	1.932(1.496, 2.495)		**<** 0.001
III	3.432 (2.026, 5.814)		**<** 0.001	1.899 (1.087, 3.317)		0.024
IV	5.225 (4.309, 6.335)		**<** 0.001	3.463 (2.820, 4.253)		**<** 0.001
Radiotherapy (*n* [%])						
No	Reference					
Yes	0.891 (0.706, 1.125)		0.333			
Chemotherapy (*n* [%])						
No	Reference					
Yes	2.508 (2.114, 2.975)		**<** 0.001			
Race (*n* [%])						
White	Reference				Reference	
Black	1.543 (0.836, 1.583)		**<** 0.001		1.513 (1.218, 1.879)	**<** 0.001
Others	0.729 (0.533, 0.995)		0.047		0.874 (0.637, 1.199)	0.403
Marital status (*n* [%])						
No	Reference					
Yes	0.904 (0.742, 1.101)		0.316			
Grade (*n* [%])						
Grade 1	Reference				Reference	
Grade 2	1.202 (0.661, 2.187)		0.546		0.947 (0.517, 1.735)	0.860
Grade 3	7.862 (4.533, 13.636)		**<** 0.001		2.903 (1.613, 5.223)	**<** 0.001
Grade 4	8.198 (4.803, 13.993)		**<** 0.001		2.855 (1.609, 5.068)	**<** 0.001
Peritoneal cytology (*n* [%])						
Negative	Reference				Reference	
Positive	5.049 (3.591, 7.098)		**<** 0.001		2.815 (1.953, 4.056)	0.049
Unknown	1.281 (1.070, 1.534)		0.007		1.201 (1.001,1.441)	**<** 0.001
Pelvic lymph node LODDS grade (*n* [%])						
**≤** 0.9542	Reference					
**≥** − 0.9542	1.337 (1.106, 1.616)		0.003			
Paraaortic LODDS (*n* [%])						
**≤** 0.4771	Reference					
**≥** − 0.4771	1.275 (1.021, 1.593)		0.032			
Tumor size (*n* [%])						
**<** 81 mm	Reference				Reference	
≥ 81 mm	2.575 (2.147, 3.090)		**<** 0.001		1.295 (1.065, 1.574)	0.009
Pathology (*n* [%])						
LE-ESS	Reference				Reference	
LMS	15.510 (8.503, 28.293)		**<** 0.001		3.977 (2.037, 7.764)	**<** 0.001
HG-ESS	17.283 (9.319, 32.053)		**<** 0.001		4.742 (2.404, 9.355)	**<** 0.001
UES	23.738 (11.890, 47.393)		**<** 0.001		5.431 (2.529, 11.662)	**<** 0.001
RMS	32.320 (13.710, 76.191)		**<** 0.001		4.165 (1.645, 10.548)	0.003
MA	8.150 (4.232, 15.696)		**<** 0.001		4.195 (2.091, 8.417)	**<** 0.001

In a comparison of the results of Cox regression and Lasso regression, age at diagnosis, FIGO stage, grade, peritoneal cytology, and tumor size were used to construct the nomogram for the following reasons: (1) we aimed to establish a model applicable to Asians, and Asian descent was a protective factor (HR = 0.874 [95% CI: 0.637, 1.199] vs. White, *p* < 0.001), with race excluded; (2) the AUC value of the model with added pathology did not show significant improvement.

### 3.3 Construction of the nomogram

**Figure 3:**
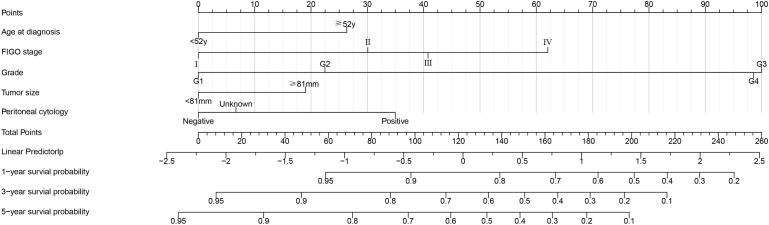
Postoperative survival nomogram established based on Lasso regression results for predicting 1-, 3-, and 5-year OS probability.

**Figure 3** shows a simple and efficient nomogram for predicting 1-, 3-, and 5-year OS probability. Factors of age at diagnosis, FIGO stage, grade, tumor size, and peritoneal cytology were used to construct a nomogram based on Lasso regression results. The tumor grade was set as a reference scale ranging from 0 to 100 because it had the largest absolute coefficient value. By adding the values above each variable, the total score was obtained. Drawing a vertical line down from the total score, the patient’s probability of survival at 1, 3, and 5 years can be obtained.

### 3.4 Evaluation and calibration of the nomogram

**Figure 4:**
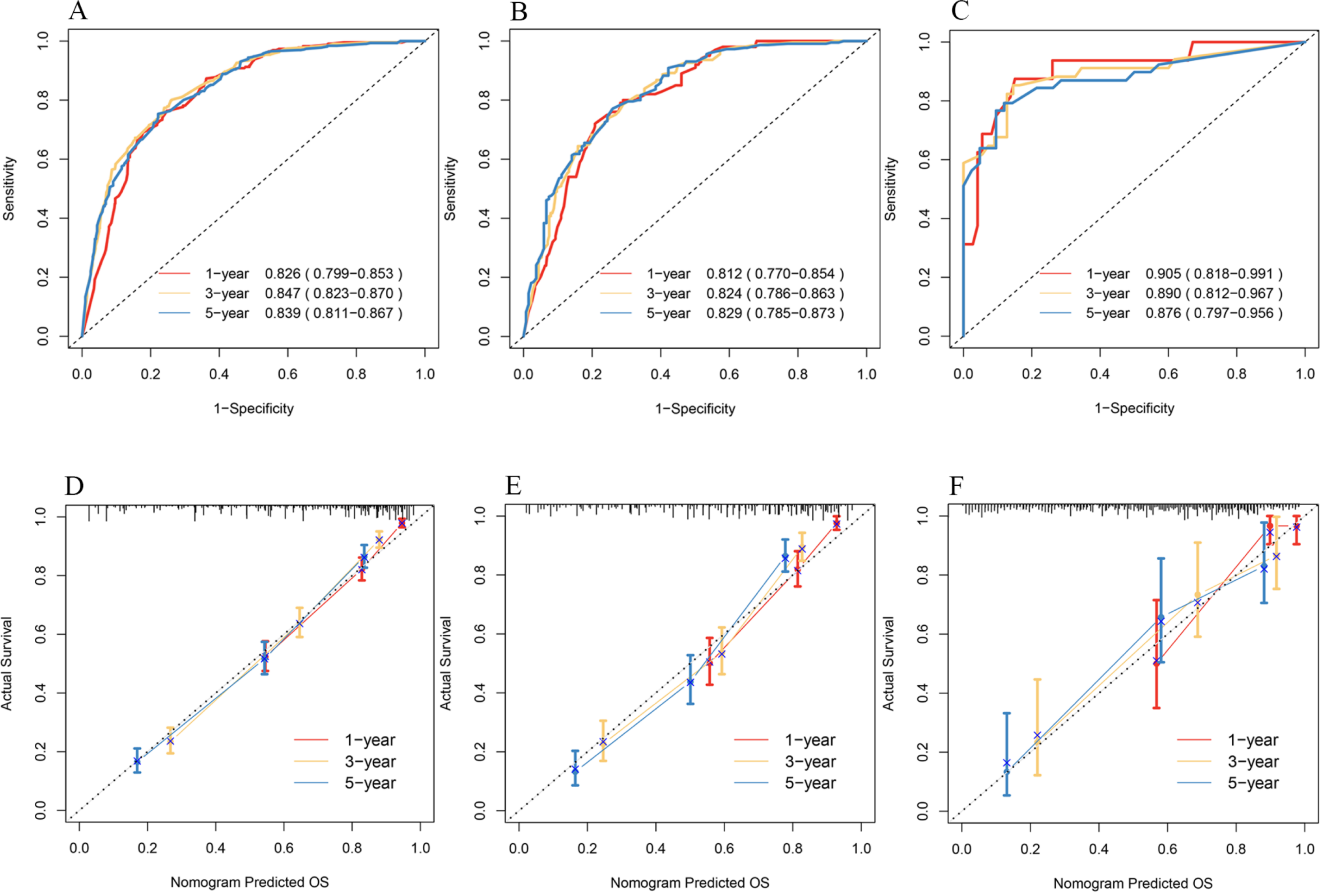
ROC curves for 1-, 3-, and 5-year OS of patients in the training set **(A)**, internal validation set **(B)**, and external validation set **(C)**. Calibration plots for 1-, 3-, and 5-year OS of patients in the training set **(D)**, internal validation set **(E)**, and external validation set **(F)**.

The C-indices in the training set, the internal validation set, and the external validation set were 0.78 [95% CI: 0.739, 0.823], 0.77 [95% CI: 0.697, 0.8255], and 0.84 [95% CI: 0.670, 0.928], respectively. The 1-, 3-, and 5-year ROC curves and AUC values were used to judge the discrimination ability of the model. In the training set (**Figure 4A**), the AUC values were 0.826, 0.847, and 0.839, respectively. The AUC values were similar in the internal validation set (**Figure 4B**). In the external validation set (**Figure 4C**), the AUC values were 0.905, 0.890, and 0.876. AUC values above 0.8 indicate strong predictive power; the external validation AUC peaked at 0.9, demonstrating excellent generalizability. Again, the calibration plots (**Figures 4D–F**) were close to the ideal 45° reference line, and the predicted values and actual results were similar.

### 3.5 Clinical usefulness

**Figure 5:**
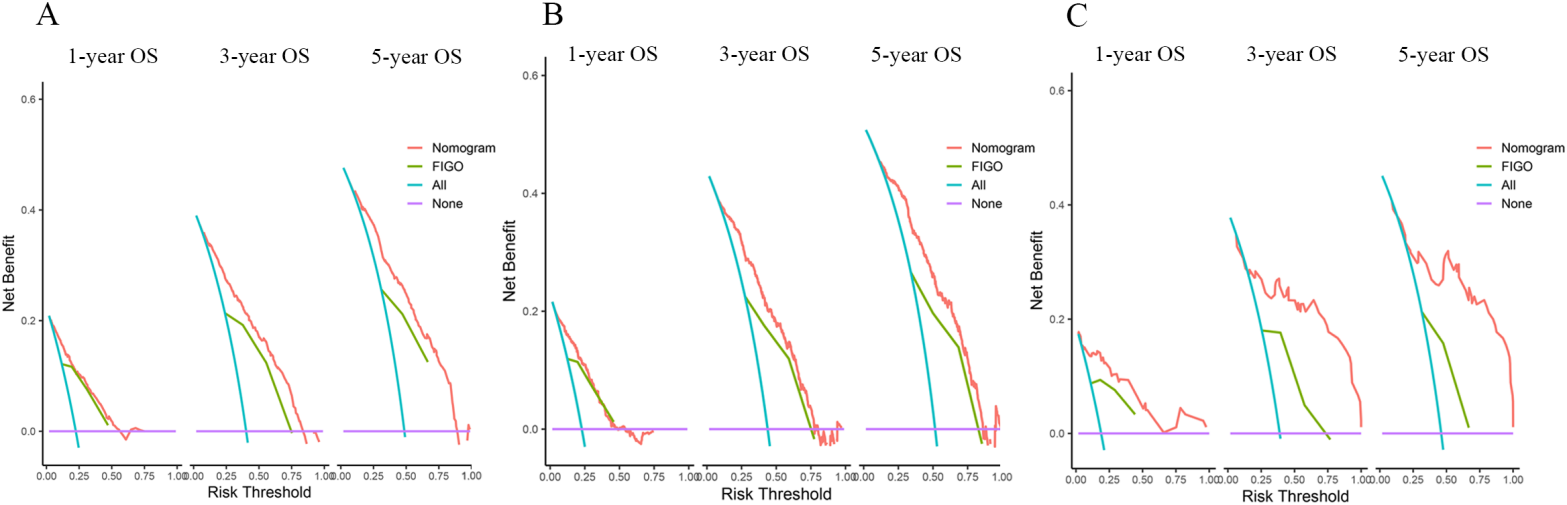
DCAs compared the nomogram with the FIGO stage for OS in the training set **(A)**, internal validation set **(B)**, and external validation set **(C)**.

DCA was a new method of evaluating alternative prognostic strategies that provided advantages over AUC. We compared the net clinical benefits of the model with FIGO staging, and the model showed clear advantages, especially in the external validation set (**Figure 5**).

### 3.6 The performance of the nomogram in prognosis stratification

An appropriate threshold is set according to the patient’s 5-year OS as an inflection point of clinical treatment strategy (9). In general, an overall 5-year relative survival > 65% is good, 35%–65% is moderate, and less than 35% is very poor (19). Patients with uterine sarcoma with good 5-year OS do not always benefit from adjuvant therapy after surgery (1). In this model, a risk-averse cut-off point of 40%/80% was used for treatment decisions based on the patient’s 5-year OS. Within the clinical prognostic nomogram we constructed, each of the five predictors was assigned a standardized point score from 0 to 100, quantifying its relative contribution to the predicted outcome. The specific points for each variable, derived from the statistical algorithm using the R software, are detailed in **Table 5**.

**Table 5:** The score for each variable in the nomogram.

Variables	Individual scores
Age at diagnosis	
**<** 52years	0
≥ 52years	26
FIGO stage	
I	0
II	30
III	41
IV	62
Grade	
Grade 1	0
Grade 2	22
Grade 3	100
Grade 4	99
Tumor size points	
**<** 81 mm	0
≥ 81 mm	19
Peritoneal cytology points	
Negative	0
Positive	35
Unknown	7

By aggregating the points from all variables, a comprehensive risk score (total points) was derived for each patient in the cohort. The results are shown in **Table 6**.

**Table 6:** Divide risk groupings based on total scores.

Group	Total score	5-Year survival probability
Low risk	≤ 55	≥ 0.8
Medium risk	− 117	0.5–0.7
High risk	**>** 117	≤ 0.4

**Figure 6:**
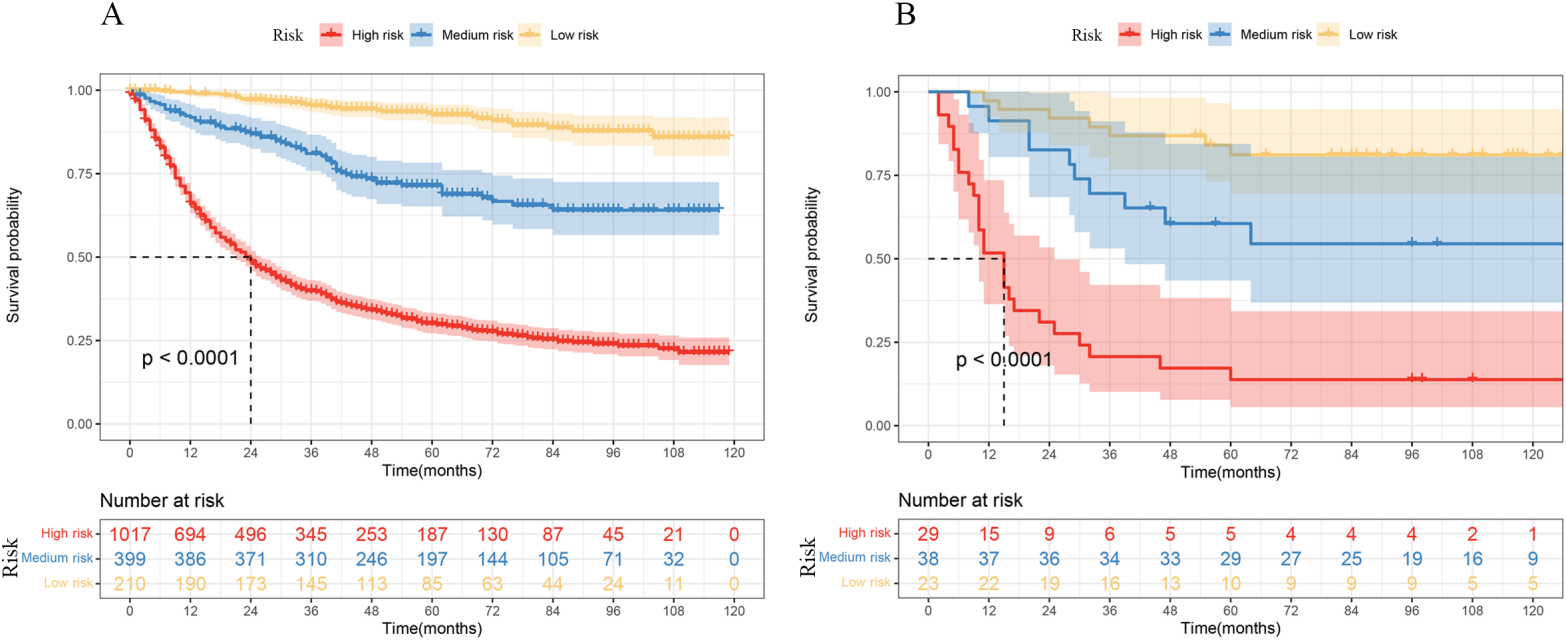
Kaplan–Meier curves of OS by risk groups based on the nomogram in the SEER data **(A)** and THAHUST data **(B)**.

All patients were divided into a high-risk group, a medium-risk group, and a low-risk group based on the tiered scores 55 and 117. The Kaplan–Meier curves and the log-rank test showed that patients with a lower total score of 55 had a higher 5-year survival probability of more than 80%, and by comparing the actual postoperative adjuvant treatment of these patients, we found that low-risk group patients did not require postoperative adjuvant therapy. Patients with a score of 55 to 117 were recommended for postoperative adjuvant treatment based on the patient’s willingness to treat. High-risk patients must undergo postoperative adjuvant therapy (*p* < 0.0001). The results are shown in **Figure 6**.

## 4 Discussion

At present, total hysterectomy or bilateral salpingo-oophorectomy (BSO) is still the standard surgical treatment for uterine sarcoma (3), and there is no uniform conclusion on the benefit of adjuvant therapy for uterine sarcoma patients after surgery, especially for patients with low-grade and early-stage disease (20–22). Even if patients are diagnosed at early stages, the prognosis is still not optimistic, and the 5-year survival rate is around 42% (1). LG-ESSs often present with long-term recurrence, and studies have shown limited benefit of adjuvant therapy, which presents a clinical challenge (23, 24). Our model supports the clinical decision to withhold adjuvant therapy in low-risk patients—e.g., a 50-year-old woman with FIGO stage II disease, tumor size of 60 mm, and grades 1–2 histology—while identifying high-risk individuals (grades 3–4) who may benefit from additional treatment.

Age at diagnosis, FIGO stage, grade, peritoneal cytology, and tumor size are readily accessible in clinical settings. We listed the scores for each variable and the corresponding hazard grouping, which is convenient for clinical application.

Postoperative adjuvant treatments, including radiotherapy and chemotherapy, were not considered protective prognostic factors. This means that uterine sarcoma is not sensitive to chemoradiotherapy and has little effect on OS. This is consistent with the results of a real-world study (4).

LODDS has been recognized in recent years as a novel prognostic factor. It has demonstrated a better prognosis than AJCC lymph node staging in a variety of cancers (17–19). However, lymphadenectomy is controversial in patients with uterine sarcoma (25, 26). Notably, our model shows that pelvic and paraaortic nodal LODDS grade was not associated with patient OS, which suggests that uterine sarcoma surgery does not require excessive lymph node dissection.

The two main limitations of this study are as follows. First, our findings may have introduced selection bias because of their retrospective nature. We excluded patients with incomplete data during the data collection process, which may have limited the generalizability or robustness of the conclusions. Second, important prognostic factors not captured in the SEER database include gene alterations (27, 28), history of uterine power morcellation (29), high parity (ten or more deliveries) (30), lymphovascular space invasion (LVSI), and hormonal therapy. A more comprehensive model of survival prognosis with optimal prognostic factors is expected to be established in the future.

## 5 Conclusions

This nomogram may help clinicians personalize adjuvant treatment decisions in uterine sarcoma, especially in low-risk Asian patients. Prospective validation is warranted.

## Data Availability

The original contributions presented in the study are included in the article/**Supplementary Material**. Further inquiries can be directed to the corresponding author.
